# Sex-specific BMI, 24-hour movement behaviors, motor skills, and executive functions in Tunisian preschool children: an observational cross-sectional study

**DOI:** 10.3389/fphys.2026.1853244

**Published:** 2026-05-20

**Authors:** Sonia Meksi, Mohamed Amine Ltifi, Waad Alfawaz, Abdulazeem Alotaibi, Mohamed Souhaiel Chelly

**Affiliations:** 1 Research Laboratory (LR23JS01), Sport Performance, Health & Society, Higher Institute of Sport and Physical Education of Ksar Said, University of Manouba, Tunis, Tunisia; 2 University of Gafsa, Higher Institute of Sport and Physical Education of Gafsa, Gafsa, Tunisia; 3 Community Health Sciences Department, College of Applied Medical Sciences, King Saud University, Riyadh, Saudi Arabia; 4 Department of Physical Education and Kinesiology, College of Education, Qassim University, Qassim, Saudi Arabia

**Keywords:** 24-hour movement behaviors, BMI, executive functions, motor skills, North Africa, observational study, preschool children

## Abstract

**Background:**

Early childhood is a critical period for motor, cognitive, and behavioral development, and the rising prevalence of overweight and obesity are associated with differences in physical activity (PA), motor skills (MS), and executive functions (EF). This study aimed to examine associations between sex-specific BMI categories, 24-hour movement behaviors (MB), MS, and EF in Tunisian preschool children.

**Methods:**

A cross-sectional observational study included 112 children aged 4–5 years (50 boys, 62 girls) from urban and rural kindergartens. Anthropometric measures classified children into sex-specific BMI categories (below normal, normal, above normal). PA, sedentary behavior (SB), and sleep were objectively assessed via accelerometry over five consecutive days. EF (inhibition, working memory) and MS (functional mobility, balance, upper and lower limb strength, manual dexterity) were evaluated using standardized and validated tests.

**Results:**

Overall, BMI showed limited associations with MB, EF, and MS, but some sex-specific associations were observed. Statistically significant differences were found for upper-body strength (girls, p = 0.003), moderate intensity PA (girls, p = 0.040), and screen-based SB (boys, p = 0.049). No statistically significant differences were observed for functional mobility, balance, lower limb strength, manual dexterity, other PA measures, sleep, or EF. Anthropometric scores were higher in BMI > normal groups, most children met MVPA and sleep recommendations, but total PA compliance was low.

**Conclusion:**

This study provides novel evidence from a North African context on sex-specific associations between BMI, MB, MS, and EF in preschool children. Findings indicate limited but domain-specific functional implications of BMI and emphasize the need for longitudinal, sex-stratified studies with larger samples to clarify developmental trajectories. However, due to the cross-sectional design, these findings reflect associations only, and no causal inferences can be drawn from these results.

## 1 Introduction

Early childhood is a critical period for physical, motor, and neurocognitive development, during which executive functions (EF), motor competence, and movement behaviors (MB) undergo rapid maturation and exert a profound influence on long term health, learning capacity, and academic readiness (National Research C and Institute of Medicine Committee on Integrating the Science of Early Childhood D, 2000; Best and Miller, 2010; Diamond, 2013; Adolph and Franchak, 2017). Disruptions during this sensitive developmental window, particularly those related to unhealthy body weight, have been associated with persistent negative effects on cognitive, motor, and metabolic health (Ng et al., 2014; Barros et al., 2021; Fernandes et al., 2022).

Childhood overweight and obesity have become major global public health concerns (Whiting et al., 2021), with rapidly increasing prevalence among preschool aged children, including in low- and middle-income countries such as Tunisia (Krupsky et al., 2020; Ltifi et al., 2025b; Ltifi et al., 2025c). Excess adiposity in early childhood is associated with reduced physical fitness, impaired motor skill development, and elevated cardiometabolic risk (Timmons et al., 2012; Palmer et al., 2020).

In addition to their well-established physical consequences, growing evidence indicates that childhood overweight and obesity are associated with alterations in brain structure and function that impair EF, including inhibitory control, impulsivity regulation, cognitive flexibility, planning, and working memory, which are essential for self-regulation, learning, and school readiness (Martí-Nicolovius, 2022). These cognitive impairments are thought to be mediated by obesity related metabolic and inflammatory processes that affect the hippocampus and prefrontal cortex, key regions involved in attention, memory, and executive control (Mamrot and Hanć, 2019; Ltifi et al., 2025a). However, findings in preschool aged children remain in consistent, partly due to methodological heterogeneity and limited use of objective measures for both physical activity (PA) and neurocognitive assessment (Mamrot and Hanć, 2019; Martí-Nicolovius, 2022; Ltifi et al., 2025a).

The 24-hour movement behavior framework emphasizes the integrated role of PA, sedentary behavior (SB), and sleep in shaping child health and development (Tremblay et al., 2017; Taylor et al., 2018; Okely et al., 2021; Ltifi et al., 2025b; Ltifi et al., 2025c). Higher levels of moderate-to-vigorous PA (MVPA) and adequate sleep duration are consistently associated with enhanced executive function and motor competence in preschool children (Barros et al., 2021; Alonso-Martínez et al., 2025), whereas excessive sedentary time, especially screen based behaviors, has been linked to poorer cognitive and motor outcomes (Diamond, 2013; Duch et al., 2013; Clark et al., 2021). Sleep also plays a crucial role in brain maturation, memory consolidation, emotional regulation, and learning processes during early childhood (Jiang, 2019; Whiting et al., 2021).

Motor Skills (MS) is another key determinant of healthy development and participation in PA. Children with overweight or obesity frequently exhibit poorer motor coordination, balance, and relative strength compared with their normal-weight peers, which may limit participation in active play and structured physical activities (Cheng et al., 2016; Barros et al., 2021; Lima et al., 2021). Moreover, evidence suggests that lower MS in early childhood is associated with reduced PA and higher adiposity over time, indicating that poor MS may contribute to decreased activity levels and increased risk of weight gain (Lima et al., 2021).

Sex differences in PA patterns, body composition, motor development, and EF emerge early in life. Boys generally display higher levels of PA, particularly in moderate to vigorous activities, and outperform girls in certain motor tasks, while girls often show earlier maturation in specific EF (Shah et al., 2020; Zorrilla-Revilla et al., 2022). Associations between body mass index (BMI), body composition, and functional performance also differ by sex, with boys and girls showing distinct patterns of fat accumulation and MS even in early childhood, supporting the need for sex-specific BMI classification when examining health and developmental outcomes (Bergqvist-Norén et al., 2022).

Evidence from early childhood research suggests that sex-related differences in growth trajectories, body composition, and neuromotor development are already observable during the preschool period, with boys and girls demonstrating distinct patterns of motor performance and physical activity engagement even before school age. Systematic reviews in preschool populations have shown that boys tend to engage in higher levels of moderate-to-vigorous physical activity, whereas girls may display earlier maturation in certain executive function domains and differences in motor skill development (Vuontela et al., 2013; Bolger et al., 2021; Terrón-Pérez et al., 2021; National-level and state-level prevalence of overweight and obesity among children, adolescents, and adults in the USA, 1990-2021, and forecasts up to 2050, 2024). These early divergences are likely influenced by a combination of biological maturation, neuromuscular development, and gendered patterns of play behavior and socialization. Furthermore, sex differences (Santiago-Rodríguez et al., 2025) in adiposity distribution and physical performance have also been reported in children under 5 years (Vuontela et al., 2013), supporting the need for sex-specific analytical approaches in this age group (Cheng et al., 2016; Martins et al., 2020; Whiting et al., 2021; Zorrilla-Revilla et al., 2022). Therefore, investigating sex-specific associations at this developmental stage is relevant to better understand whether weight status differentially affects motor and cognitive outcomes in early childhood (Santiago-Rodríguez et al., 2025).

Despite increasing global interest in early childhood MB and obesity (Kurspahić-Mujčić and Mujčić, 2020), data from North Africa and Tunisia remain scarce. Most studies in preschool children rely on parent reported PA and SB, which are prone to measurement bias and may underestimate true variability (Ltifi et al., 2025a). Moreover, few studies have simultaneously examined BMI, objectively measured 24-hour MB, MS, and EF using a sex-specific analytical framework.

Following the World Health Organization (WHO) guidelines for children under 5 years, which provide evidence-based recommendations on PA, SB, and sleep to promote optimal health and development, it is crucial to understand how young children’s MB relate to their physical and cognitive functioning (de Onis et al., 2007; de Onis et al., 2009; WHO Guidelines Approved by the Guidelines Review Committee, 2019; Okely et al., 2021). These guidelines advise on the amount of time children should spend being physically active or sleeping, and the maximum recommended time for screen based sedentary activities or restrained behaviors, in order to support overall health and wellbeing. Adherence to these recommendations has been associated with improved MS, cognitive development, and long-term health outcomes in early childhood (de Onis et al., 2007; de Onis et al., 2009; WHO Guidelines Approved by the Guidelines Review Committee, 2019; Okely et al., 2021).

Therefore, in line with these international recommendations, the aim of this observational cross-sectional study was to examine the associations between sex-specific BMI categories, 24-hour MB measured by accelerometry, MS, and EF in Tunisian preschool children under 5 years of age.

We hypothesized that BMI categories would be significantly associated with variations in daily MB, MS performance, and EF outcomes, and that these associations would differ according to sex. Specifically, we expected children with BMI above the normal range to demonstrate lower motor performance, reduced compliance with PA recommendations, higher SB, and less favorable EF scores compared with their normal-weight peers.

Although previous research (Carter et al., 2011; Tremblay et al., 2011; Barnett et al., 2016; Draper et al., 2020; Bai et al., 2021; Barros et al., 2021; Fernandes et al., 2022) has reported associations between BMI, MS, and cognitive functioning, findings remain inconsistent in preschool-aged populations, particularly in low- and middle-income countries. To the best of our knowledge, no prior study in the North African context has simultaneously examined BMI, objectively measured 24-hour MB, MS, and EF in young children.

By integrating objective movement data with standardized cognitive and motor assessments, the present study provides a comprehensive and developmentally sensitive perspective on how weight status relates to both physical and cognitive functioning in early childhood, thereby contributing novel evidence from a middle-income North African setting.

## 2 Materials and methods

### 2.1 Participants

This observational cross-sectional study included 112 preschool children (50 boys and 62 girls) aged 4 to 5 years (mean ± SD: 4.10 ± 0.58 years; median: 4.13 years). Participants were recruited from five kindergartens located in urban (El Manar and El Omrane) and rural (Cité Zahrouni and Séjoumi) regions of the Greater Tunis area, the capital region of Tunisia, selected randomly to ensure a diverse and representative sample in terms of sociodemographic and environmental contexts. A convenience sampling approach was used based on the voluntary participation of kindergartens and parents. All eligible children aged 4–5 years within each participating kindergarten were invited to take part in the study. A total of 28 children and their parents declined participation, resulting in an overall participation rate of approximately 81%. The sample was designed to increase heterogeneity rather than to achieve national representativeness.

Children were eligible if they were aged between 4 and 5 years and had written parental or legal guardian consent. Exclusion criteria included any medical condition, physical disability, developmental disorder, or other conditions likely to influence MB, MS, or EF. Although urban–rural differences are well documented (Ltifi et al., 2025b), data from both settings were combined to increase statistical power while minimizing confounding. The final sample could reflect the typical sociodemographic characteristics of preschool children in these regions, though it is not fully nationally representative.

However, despite including both urban and rural settings, the sample remains regionally restricted to Greater Tunis and may not fully capture the sociodemographic and cultural diversity of the entire Tunisian population or the broader North African region. It should be noted that preschool attendance in Tunisia is not compulsory.

Sex-specific BMI distribution was as follows:

- Boys (n = 50): BMI < normal = 1; BMI = normal = 31; BMI > normal = 18.
- Girls (n = 62): BMI < normal = 4; BMI = normal = 38; BMI > normal = 20.

This sex-specific classification allowed comparisons within each sex across BMI categories, providing more precise insights into the associations between BMI, MB, MS, and EF. However, no *a priori* power analysis was conducted to determine the required sample size. Given the stratification by sex and BMI categories, some subgroups were small, which may have reduced the statistical power to detect small-to-moderate associations.

### 2.2 Procedure

All measurements were conducted within the kindergarten setting by a trained research teacher and two research assistants, following standardized data collection protocols to ensure reliability and consistency.

The order of assessments was standardized across all kindergartens. On the first day, children were fitted with the accelerometer and anthropometric measurements were performed. On the second day, gross motor skill tests were administered in the following order: functional mobility (Supine Timed Up and Go), postural balance (one-leg standing), upper body strength (handgrip), and lower body strength (standing long jump). On the third day, fine motor dexterity (9-Hole Pegboard Test) and the inhibition task (Mr. Ant) were administered. On the fourth day, the working memory task (Go/No-Go) was conducted, and accelerometers were removed at the end of the day, which corresponded to Friday. After each test, children were given sufficient rest while other participants completed their assessments to minimize fatigue and potential learning effects.

Anthropometric measurements, motor skill assessments, and executive function tasks were administered individually to each child. Accelerometers were distributed to participants and worn for five consecutive days during their usual daily activities to objectively capture MB.

Parent and kindergarten questionnaires were completed through face-to-face interviews at times convenient for families and school staff. All questionnaires and instructions were provided in Arabic to ensure clear understanding and accurate reporting.

### 2.3 Anthropometry and sex-specific BMI classification

#### 2.3.1 Anthropometry

Height was measured to the nearest 0.1 cm using customizable and repositionable adhesive measuring tapes, following standardized anthropometric procedures. Children stood barefoot in an upright position, with their head aligned according to the Frankfurt plane. Each height measurement was performed twice, and if the difference between the two measurements exceeded 0.5 cm, a third measurement was taken. The mean of the two closest values was used for analysis.

Body weight was measured to the nearest 0.1 kg using a calibrated SECA 750 Viva scale (SECA, Hamburg, Germany), with children wearing light clothing and no shoes. Two measurements were recorded, and if the difference exceeded 0.25 kg, a third measurement was conducted. The average value was used for subsequent analyzes. BMI was calculated as weight (kg) divided by height squared (m²). Height-for-age (HAZ), weight-for-age (WAZ), and BMI-for-age (BAZ) z-scores were computed according to WHO growth reference standards (WHO child growth standards based on length/height, weight and age, 2006).

#### 2.3.2 BMI classification and sex-specific categorization

standards BMI Classification and Sex-Specific Categorization (WHO child growth standards based on length/height, weight and age, 2006; Okely et al., 2021). Initially, participants were categorized as: severely thin (< −3 SD), thin (−3 to < −2 SD), normal weight (−2 to +1 SD), at risk of overweight (> +1 to +2 SD), overweight (> +2 to +3 SD), or obese (> +3 SD).

For statistical analyzes, categories were consolidated into three groups (WHO child growth standards based on length/height, weight and age, 2006; WHO Guidelines Approved by the Guidelines Review Committee, 2019; Okely et al., 2021):

- BMI below normal (severe thinness and thinness).
- BMI normal.
- BMI above normal (risk of overweight, overweight, and obesity).

This classification adheres to WHO guidelines for children aged 0–60 months (WHO Anthro) and 61 months to 19 years (WHO AnthroPlus) (Okely et al., 2021), allowing a standardized assessment of weight status adjusted for age and sex (Breastfeeding in the WHO multicentre growth reference study, 2006; WHO child growth standards based on length/height, weight and age, 2006; Okely et al., 2021). For analytical purposes, the three-category grouping was applied within each sex, enabling within-sex comparisons and ensuring robust evaluation of associations between BMI, 24-hour MB, MS, and EF in preschool children.

#### 2.3.3 Physical activity, sedentary behavior, and sleep

PA, SB, and sleep were objectively measured using ActiGraph GT3X accelerometers (ActiGraph LLC, Pensacola, Florida, USA) worn on the right hip for five consecutive days. Accelerometers were set at a sampling frequency of 30 Hz, and raw data were aggregated into 15-second epochs using a low-frequency filter. Data processing was performed using ActiLife software (version 6.1.2.1). A valid day was defined as a minimum of 24 hours of recorded data, including at least 6 hours of valid waking wear time.

Non-wear periods were defined as 20 consecutive minutes or more of zero counts and were excluded from analyzes. Sleep periods were identified from accelerometer recordings and verified using parental reports and activity logs, and were excluded from PA and SB analyzes.

Activity intensity thresholds were defined based on established standard cut-points (Pate et al., 2006; Pate et al., 2015), with SB was defined as less than 800 counts per minute, light-intensity PA ranging from 800 to 1679 counts per minute, moderate-intensity PA ranging from 1680 to 3367 counts per minute, and vigorous-intensity PA defined as 3368 counts per minute or higher (Smith et al., 2019). These thresholds allowed for a precise classification of MB and facilitated the objective quantification of daily PA patterns in preschool children.

### 2.4 Executives functions

Executives functions were assessed using the Early Years Toolbox (Smith et al., 2000; Spencer, 2021), with a specific focus on inhibition and visuospatial working memory. The Early Years Toolbox has demonstrated good reliability and validity in international studies with preschool children, and has been widely used across different cultural and linguistic contexts in preschool populations (Ltifi et al., 2025b; Ltifi et al., 2025c; Ltifi et al., 2025a). Each task lasted approximately 10 minutes and included a standardized practice phase at the beginning to familiarize children with the procedures. The original French versions of the tasks were translated verbatim into Arabic by a field worker, who was also a physical education teacher, using a forward translation method to ensure accurate adaptation of the content. The translated version was reviewed by the research team to ensure linguistic consistency and conceptual equivalence with the original instrument (Okely et al., 2021; Ltifi et al., 2025a).

In addition, cultural adaptation was undertaken to ensure that instructions and vocabulary were appropriate for Tunisian preschool children, particularly in terms of language simplicity and task comprehension. Basic checks of understanding were conducted during the initial administration phase to confirm that children were able to follow the instructions and perform the tasks as intended. No major difficulties were observed during the administration of the different tasks (inhibition and working memory), with all children being able to complete the tasks without interruption or exclusion. Good engagement was observed in association with generally cooperative behavior throughout the assessments, with an adequate level of motivation supported by the evaluators. This Arabic version is currently undergoing validation among Tunisian preschool children and has been employed in previous studies within the Tunisian context (Ltifi et al., 2025b; Ltifi et al., 2025a), confirming its acceptability and comprehensibility for this population. All EF assessments were administered individually in a quiet kindergarten setting by trained research assistants.

### 2.5 Gross and fine motor skills

Motors skills were assessed using selected tests from the NIH Toolbox (Gershon et al., 2013; Okely et al., 2021) in a spacious classroom environment (Okely et al., 2021), allowing children to perform each task safely and comfortably. These assessments have established reliability and validity in international studies of preschool children, supporting their use for evaluating gross and fine MS. The assessment included both gross MS and fine MS, described below (Draper et al., 2020; Munambah et al., 2021).

#### 2.5.1 Functional mobility: supine timed up and go test

Functional mobility was evaluated using the supine Timed Up and Go test. Children began lying on their backs behind a marked line, then stood up as quickly as possible, ran to touch a target located 3 meters away, and returned to the starting position. Each participant completed one practice trial followed by two test trials, and the mean performance time was used for analysis. This test assesses mobility, speed, and coordination (Draper et al., 2020; Okely et al., 2021; Zarghani et al., 2024; Ltifi et al., 2025a).

#### 2.5.2 Postural stability: one-leg standing balance test

Postural control was assessed using the one-leg standing balance test. Children stood on one leg with their arms at their sides while lifting the opposite foot off the ground. The trial ended if the standing foot shifted or the free leg was wrapped around it. Each leg was tested for a maximum of 30 seconds, and the mean duration across both legs was used for analysis. This test evaluates static balance and lower-limb postural control (Draper et al., 2020; Munambah et al., 2021; Zarghani et al., 2024).

#### 2.5.3 Upper body strength: hand grip dynamometer

Upper body strength was measured using a hand grip dynamometer (TKK 5825, Grip-A). Children were instructed to squeeze the device with maximal force for at least 3 seconds without touching their body. Each child completed one practice trial and two recorded trials per hand, and the highest value or mean value was used for analysis. This test assesses isometric handgrip strength, a key indicator of overall upper body muscular strength (Mathiowetz et al., 1986; Draper et al., 2020; Munambah et al., 2021; Zarghani et al., 2024).

#### 2.5.4 Lower body strength and mobility: standing long jump

Lower body strength and explosive power were evaluated using the standing long jump. Children stood behind a marked line and jumped forward as far as possible using both feet. Following a practice jump, two trials were recorded, and the mean jump distance was used for analysis. This test assesses leg muscle strength, coordination, and mobility (Draper et al., 2020; Munambah et al., 2021; Zarghani et al., 2024).

#### 2.5.5 Fine motor skills: 9-hole pegboard test

Fine motor dexterity was assessed using the 9-Hole Pegboard Test. Children were instructed to place and remove nine pegs one by one as quickly as possible. Timing started when the first peg was touched and stopped when the last peg was removed. The total completion time (seconds) was recorded for analysis. This test has demonstrated high reliability and validity for evaluating fine MS in children (Smith et al., 2000) and provides insight into manual dexterity, hand-eye coordination, and precision (Draper et al., 2020; Munambah et al., 2021; Zarghani et al., 2024).

### 2.6 Statistical analysis

No *a priori* sample size calculation was performed, as this study adopted a pragmatic observational cross-sectional design. The sample size was determined based on feasibility and participant availability during the data collection period. All analyzes were conducted using SPSS Statistics for Windows, version 26.0. Continuous variables are presented as mean ± standard deviation (SD), and categorical variables as counts and percentages.

Comparisons across BMI categories were performed separately for boys and girls to allow sex-specific analyzes. For continuous variables, Mann-Whitney U test was used for comparisons between two BMI categories in boys (BMI = normal vs. BMI > normal), given that the BMI < normal category was excluded from the statistical analysis due to insufficient sample size (n = 1). For girls, where three BMI categories were compared (BMI < normal, BMI = normal, and BMI > normal), one-way ANOVA followed by Tukey’s *post hoc* test was applied for pairwise comparisons. Prior to ANOVA, homogeneity of variances was verified using Levene’s test for all continuous variables. Due to the small sample size, effect sizes were calculated to supplement p-values. For Mann-Whitney U tests, the rank correlation “r” was used, while for Kruskal-Wallis tests eta-squared (η²_H_) was applied. Effect sizes were interpreted as small (0.1), medium (0.3), or large (0.5) according to Cohen’s criteria. However, for categorical variables, Pearson’s chi-square test was used for comparisons involving two groups, while Fisher’s exact test was applied for comparisons involving three groups. When a significant overall effect was observed for continuous variables, pairwise comparisons were conducted, with Bonferroni correction applied to adjust for multiple testing.

To address the limitations of univariate analyzes and preserve the statistical information contained in BMI-for-age z-scores (BAZ), a series of multiple regression analyzes were conducted treating BAZ as a continuous variable. For continuous outcomes, including inhibition, working memory, functional mobility, postural steadiness, lower limb strength, upper limb strength, and dexterity, multiple linear regression models were applied. Each model included BAZ as the primary predictor of interest, with age and sex entered simultaneously as covariates to control for potential confounding effects. Model fit was assessed using the coefficient of determination (R²) and adjusted R², and overall model significance was evaluated using the F-test from the ANOVA table. Unstandardized (B) and standardized (β) regression coefficients are reported alongside 95% confidence intervals and corresponding p-values for each predictor.

This sex-specific classification enabled within sex comparisons across BMI categories and ensured accurate assessment of the relationships between BMI, 24-hour MB, MS, and EF.

Children were considered to meet the PA guidelines if they accumulated an average of ≥ 180 min/day of total PA, including ≥ 60 min/day of moderate-to-vigorous PA (MVPA), as measured by accelerometry. Accelerometer data were processed using ActiLife software (version 6.1.2.1). Non-wear time was defined as ≥ 20 consecutive minutes of zero counts and excluded from analyzes. A valid day required at least 24 hours of data, including a minimum of 6 hours of valid waking wear time.

Sleep periods were identified from accelerometer data and confirmed via parental reports and activity logs, and were excluded from the PA and SB analyzes. Compliance with sedentary screen time (SST) recommendations was defined as ≤ 60 min/day, and compliance with sleep duration recommendations was defined as 10–13 hours per 24-hour period, according to parent-reported data. These definitions adhere to the WHO Guidelines on PA, SB, and Sleep for Children Under 5 Years (Breastfeeding in the WHO multicentre growth reference study, 2006; WHO Guidelines Approved by the Guidelines Review Committee, 2019; Okely et al., 2021). The level of statistical significance was set at p < 0.05.

In order to retain statistical information and prevent potential instability associated with severely unbalanced group sizes, BMI-for-age Z-scores (BAZ) were treated as a continuous variable rather than divided into discrete groups in accordance. Age and sex were simultaneously entered as factors in multiple linear regression analyzes to investigate the separate relationships between BAZ and motor and executive function results. Adjusted coefficients of determination (Adj. R²) and unstandardized regression coefficients (B) with 95% confidence intervals are presented. The threshold for statistical significance was fixed at p < 0.05.

## 3 Results

**Table 1** presents the anthropometric characteristics and 24-hour MB of preschool children according to BMI categories using a sex-specific classification. Significant differences were observed in several anthropometric parameters.

**Table 1:** Anthropometric characteristics and movement behaviors stratified by BMI categories by sex.

			Anthropometric characteristics							
		*Boys (n* = *50)*			*Girls (n* = *62)*					
Variables	All (n = 112)	*BMI = normal* *(n = 31)*	*BMI > normal* *(n = 18)*	*P* *(Effect size)*	*BMI < normal* *(n = 4)*	*BMI = normal (n = 38)*	*BMI > normal* *(n = 20)*	*95% CI*	*ANOVA-1* *P (Effect Size)*	*Post hoc* *p*
Age (years)	4.10±0.58	4.09±0.53	4.00±0.63	0.575 (0.08)	4.39±0.68	4.27±0.57	3.81±0.52	[-0.58;0.82] € [-0.15;1.31] Π [0.09;0.83] β	0.01 (0.144)	0.914 € 0.148 Π ***0.011 β***
Weight (kg)	18.60± 2.93	18.08 ± 2.02	20.79 ± 2.71	**0.001** (0.48)	12.93 ± 2.09	18.03 ± 2.36	19.94 ± 3.05	[-8.37;-1.83] € [-10.42;-3.60] Π [-3.62;-1.90] β	**<0.001** (0.303)	**0.001 €** **<0.001Π** **0.026 β**
Height (cm)	107.38±6.76	108.74±6.71	106.92±5.83	0.263 (0.16)	103.16±8.86	108.76±6.95	104.19±6.00	[-14.15;2.95] € [-9.93;7.89] Π [0.07;9.07] β	0.03 (0.110)	0.265 € 0.959 Π **0.045 β**
BMI (kg/m²)	16.12±2.00	15.28±0.94	18.16±1.64	**<0.001** (0.80)	12.09±0.35	15.21±1.06	18.29±1.22	[-4.49;-1.74] € [-7.63;-4.76] Π [-3.79;-2.35] β	**<0.001** **(0.730)**	**<0.001 €** **<0.001 Π** **<0.001 β**
HAZ	0.85±1.20	0.66±0.87	1.85±1.04	**<0.001** (0.54)	-2.06±0.83	0.51±0.83	1.65±0.72	[-3.57;-1.55] € [-4.75;-2.65] Π [-1.66;-0.60] β	**<0.001** **(0.570)**	<**0.001 €** **<0.001 Π** **<0.001 β**
WAZ	0.89±1.26	1.15±1.32	0.93±1.43	0.431 (0.11)	-0.52±1.18	0.97 ± 1.21	0.70±0.95	[-2.90;-0.05] € [-2.70;0.26] Π [-0.48;1.01] β	**0.04** **(0.098)**	**0.040 €** 0.129 Π 0.667 β
BAZ	0.48±1.37	-0.06±0.71	1.94±1.00	<0.001 (0.83)	-2.67±0.34	-0.07±0.76	1.85±0.64	[-3.48;-1.70] € [-5.44;-3.59] Π [-2.39;-1.45] β	**<0.001** **(0.752)**	**<0.001 €** **<0.001 Π** **<0.001 β**
			*Physical activity, sedentary behavior and sleep*							
		Boys (n = 42)				Girls (n = 46)				
Variables	All (n = 89)	BMI = normal (n = 28)	BMI > normal (n = 14)	P (Effect size)	BMI < normal (n = 3)	BMI = normal (n = 32)	BMI > normal (n = 11)	95% CI	ANOVA-1 P (Effect Size)	Post hoc p
LPA (min/day)	88.3±24.7	92.7±25.2	93.1±18.9	0.790 (-0.023)	82.6±19.3	81.5±23.6	93.6±32.8	[-36.75;38.97] € [-51.76;29.92] Π [-33.94;9.89] β	0.416 (0.040)	0.997 € 0.794 Π 0.385 β
MPA (min/day)	61.8±23.7	68.3±26.9	65.7±20.3	0.968 (-0.025)	64.4±29.0	52.6±17.7	62.6±30.0	[-20.14;43.74] € [-32.60;36.30] Π [-28.43;8.54] β	0.340 (0.049)	0.645 € 0.991 Π **0.040 β**
VPA (min/day	18.8±12.9	24,0±16.1	20.5±10.8	0.650 (-0.020)	18.0±11.3	14.5±7.8	17.2±14.8	[-11.20;18.17] € [-15.00;16.68] Π [-11.15;5.86] β	0.676 (0.018)	0.834 € 0.991 Π 0.732 β
MVPA (min/day)	80.3±34.9	92.2±41.4	86.2±29.9	0.894 (-0.025)	82.4±40.2	67.2±23.7	79.8±43.0	[-28.93;59.49] € [-45.00;50.38] Π [-38.18;13.00] β	0.399 (0.042)	0.681 € 0.990 Π 0.463 β
TPA (min/day)	168.6±55.1	185.0±62.5	179.3±43.2	0.936 (-0.025)	165.1±58.1	148.7±45.1	173.3±67.0	[-59.32;92.10] € [-89.90;73.44] Π [-68.44;19.20] β	0.382 (0.044)	0.859 € 0.968 Π 0.369 β
SB (min/day)	628.6±56.5	619.7±64.3	612.6±44.4	0.936 (-0.007)	647.7±74.7	646.2±48.6	611.7±59.6	[-75.86;78.89] € [-47.47;119.47] Π [-10.30;79.28] β	0.177 (0.077)	0.999 € 0.552 Π 0.160 β
SST (min/day) a	70.8±33.3	67.65±28.49	70.78±36.09	0.834 (0.03)	60.25±13.48	71.3±35.1	75.2±38.6	[-0.56.01;33.82] € [-61.75;31.85] Π [-27.46;19.75] β		0.824 € 0.724 Π 0.919 β
Sleep (min/day) a	640.4±53.5	648.0±43.0	639.6±62.8	0.442 (0.11)	596.5±18.9	635.7±55.5	645.8±58.5	[-108.95;30.63] € [-122.02;23.42] Π [-46.82;26.53] β		0.374 € 0.241 Π 0.785 β

The BMI < normal category for boys (n = 1) was excluded from the statistical analysis due to insufficient sample size. Abbreviations : BAZ, BMI-for-age z-score; BMI, body mass index; HAZ, height-for-age z-score; LPA, light-intensity physical activity; MPA, moderate-intensity physical activity per day (in minutes); MVPA, moderate-to-vigorous-intensity physical activity; SB, SST, sedentary screen time; TPA, total physical activity; VPA, vigorous-intensity physical activity per day (in minutes); WAZ, weight-for-age z-score. €: P-value or CI between BMI < normal and BMI = normal; π: P-value or CI between BMI < normal and BMI > normal; β: P-value or CI between BMI = normal and BMI > normal. CI : Confidence interval.

Bold values indicate statistical significance at P < 0.05. Differences between girls were tested using one-way ANOVA followed by Tukey's post hoc test for pairwise comparisons, Mann-Whitney or Kruskal–Wallis selected automatically by SPSS based on data characteristics (boys). Analytical sample: a n = 89, Boys (n = 43): BMI < normal = 1, BMI = normal = 28, BMI > normal = 14; Girls (n = 62): BMI < normal = 4, BMI = normal = 38, BMI > normal = 20.

### 3.1 Anthropometric characteristics and BMI

A total of 112 preschool children (50 boys and 62 girls) were included. Subgroup sizes were uneven across BMI categories (boys: BMI < normal = 1, BMI = normal = 31, BMI > normal = 18; girls: BMI < normal = 4, BMI = normal = 38, BMI > normal = 20), which should be considered when interpreting the findings. Boys with BMI < normal (n = 1) were excluded from inferential analyzes due to small sample size. Age did not differ significantly across BMI categories for boys (p = 0.575). In contrast, a significant association was found in the girls’ group between normal BMI and above-normal BMI (p = 0.011) (**Table 1**). Significant differences were found between groups in each sex-specific BMI distribution for both boys and girls. Height differences were generally non-significant, except between BMI = normal and BMI > normal in girls (p = 0.045).

As expected, BMI, height-for-age z-score (HAZ), and BMI-for-age z-score (BAZ) were significantly higher in BMI > normal groups for both boys (BMI: 18.16 ± 1.64 kg/m², HAZ: 1.85 ± 1.04, BAZ: 1.94 ± 1.00) and girls (BMI: 18.29 ± 1.22 kg/m², HAZ: 1.65 ± 0.72, BAZ: 1.85 ± 0.64) compared with BMI = normal groups (all p < 0.001). Weight-for-age z-score (WAZ) differed significantly only in girls (p = 0.040) between BMI < normal and BMI = normal.

### 3.2 24-hour movement behaviors

Analyses of PA and SB included 89 children (boys n = 43, girls n = 46). The BMI < normal category for boys (n = 1) was excluded from the statistical analysis due to insufficient sample size reducing the total number of boys (n = 42). Light-intensity PA (LPA), vigorous-intensity PA (VPA), moderate-to-vigorous PA (MVPA), total PA (TPA), SB, SST, and sleep duration did not differ significantly across sex-specific BMI distribution. Moderate-intensity PA was slightly lower in girls with BMI = normal compared with BMI > normal (53 ± 17.7 vs 63 ± 30.0 min/day, p = 0.040).

**Table 2** presents the proportion of preschool children meeting 24-hour movement guidelines stratified by BMI categories and sex. Overall, a majority of children achieved the recommended ≥60 min/day of moderate-to-vigorous PA (MVPA) and 10–13 h/day of sleep, with no significant differences observed across BMI groups for either boys or girls. Compliance with total PA (TPA) and the combined MVPA + TPA recommendations was generally low to moderate and did not differ significantly according to BMI. Notably, SST (SST ≤ 60 min/day) differed significantly among boys, with a lower proportion of children in the BMI > normal group meeting the guideline compared with BMI = normal (p = 0.049). No significant differences were observed among girls for any of the MB. The proportion of children meeting all five recommendations was low across sexes, without significant variation between BMI categories.

**Table 2:** Number and proportion of preschool children meeting 24-hour movement guidelines stratified by BMI categories by sex.

Variables		Boys (n = 43) (48.31%)			Girls (n = 46) (51.68%)			
	Total n (%)	BMI = normal (n = 28) (12.04%)	BMI > normal (n = 14) (32.56%)	chi-square P	BMI < normal (n = 3) (6.52%)	BMI = normal (n = 32) (69.56%)	BMI > normal (n = 11) (23.91%)	ANOVA P
≥ 60 min/day of MVPA, n = 89	68 (76.4%)	23 (82.1%)	11 (78.6%)	1.000	2 (66.7%)	22 (68.7%)	9 (81.8%)	0.864
≥ 80 min/day of TPA, n = 89	36 (40.5%)	14 (50%)	8 (57.14%)	0.662	1 (33.3%)	9 (28.1%)	4 (36.4%)	0.867
≥ 60 min/day of MVPA and ≥ 180 min/day of TPA, n = 89	36 (40.4%)	14 (50%)	8 (57.14%)	0.662	1 (33.3%)	9 (28.1%)	4 (36.4%)	0.867
≤ 60 min/day of SST, a n = 112	59 (52.7%)	21 (67.7%)	7 (38.9%)	**0.049**	2 (50%)	17 (44.7%)	11 (55%)	0.838
10–13 h/day of sleep, a n = 112	91 (81.3%)	26 (83.9%)	16 (88.9%)	0.628	4 (100%)	30 (78.9%)	15 (75%)	0.697
Meeting all 5 recommendations, a n = 89	16 (18.0%)	8 (25.8%)	2 (1.1%)	0.306	0 (0%)	3 (7.9%)	3 (15%)	0.329

The BMI < normal category (n = 1) was excluded from the statistical analysis due to insufficient sample size and is reported for descriptive purposes only. .

Abbreviations: MVPA, moderate-to-vigorous-intensity physical activity; SST, sedentary screen time; TPA, total physical activity. .

Analytical sample: a n = 112, Boys (n = 50): BMI < normal = 1; BMI = normal = 31; BMI > normal = 18. Girls (n = 62): BMI < normal = 4; BMI = normal = 38; BMI > normal = 20. Differences between boys were tested using Pearson chi-square tests. Group comparisons between girls were performed using the Fisher's exact test. Bold values indicate statistical significance at P < 0.05.

**Table 3** presents the outcomes of EF as well as gross and fine MS among preschool children, stratified by sex-specific BMI categories. No significant differences were observed across BMI groups for inhibition or working memory in either boys or girls (all p > 0.05), indicating that no statistically significant associations were observed between weight status and these cognitive domains in this sample, although limited statistical power may have reduced the ability to detect subtle effects. Measures of functional mobility, postural steadiness, lower body strength, and upper body strength did not differ significantly overall between BMI groups. However, a significant difference was detected in girls for upper body strength between the BMI = normal and BMI > normal groups (p = 0.003), with higher BMI in girls being associated with lower performance. However, this finding should be interpreted cautiously given the relatively small subgroup sizes. Manual dexterity, assessed via the 9-Hole Pegboard Test, showed no significant differences across BMI categories in either sex, suggesting that fine MS are not associated with weight status in this cohort.

**Table 3:** Executive function and gross and fine motor skill outcomes of preschool children stratified by BMI categories by sex (mean ± SD).

			Boys (50)		Girls (62)							
Variables	Total (n = 112)	BMI = normal (n= 31)	BMI > normal (n= 18)	P *(Effect size)*	BMI < normal (n=4)		BMI = normal (n=38)		BMI > normal (n= 20)	*95% CI)*	*ANOVA-1* *P (Effect Size)*	Post hoc P
Inhibition	0.67 ± 0.24	0.63 ± 0.24	0.63 ± 0.28	0.975 (0.00)	0.65 ± 0.25		0.71 ± 0.24		0.65 ± 0.22	[-0.35;0.24] € [-0.31;0.30] Π [-0.10;0.21] β	0.664 (0.014)	0.891 € 1.00 Π 0.671 β
Working memory	1.89 ± 0.74	1.92 ± 0.83	1.89 ± 0.81	0.917 (0.01)	1.75 ± 0.74		1.97 ± 0.72		1.70 ± 0.59	[-1.08;0.63] € [-0.84;0.94] Π [-0.17;0.72] β	0.330 (0.037)	0.806 € 0.990 Π 0.319 β
Functional mobility (s)	5.30 ± 1.41	4.58 ± 1.18	4.83 ± 1.72	0.864 (0.02)	5.00 ± 0.82		5.08 ±1.36		5.00 ± 1.21	[-1.71;1.56] € [-1.70;1.70] Π [-0.78;0.94] β	0.973 (0.001)	0.993 € 1.00 Π 0.973 β
Postural steadiness (s)	12.60 ± 7.12	16.19 ± 9.54	14.94 ± 10.37	0.454 (0.11)	13.50 ± 12.50		16.82 ±7.88		13.65 ± 9.41	[-14.29;7.66] € [-11.58;11.28] Π [-2.60;8.93] β	0.376 (0.033)	0.749 € 0.999 Π 0.390 β
Lower body strength (cm)	56.08 ± 20.80	62.32 ± 20.46	58.83 ± 23.66	0.611 (0.07)	68.25 ± 36.80		58.47 ± 20.03		48.95 ± 15.19	[-15.33;34.89] € [-6.86;45.46] Π [-3.67;22.72] β	0.108 (0.073)	0.620 € 0.187 Π 0.201 β
Upper body strength (kg)	7.24 ± 2.52	8.68 ± 2.67	8.83 ± 3.23	1.00 (0.00)	6.63 ± 3.28		8.79 ± 2.50		6.53 ± 2.03	[-5.20;0.86] € [-3.07;3.21] Π [0.66;3.86] β	0.003 (0.179)	0.207 € 0.997 Π **0.003** β
Dexterity (s)	40.94 ± 9.75	37.84 ± 8.96	40.11 ± 9.49	0.412 (0.12)	37.50 ± 9.11		35.08 ± 9.20		38.50 ± 9.84	[-9.47;14.31] € [-13.39;11.39] Π [-9.67;2.83] β	0.413 (0.030)	0.877 € 0.979 Π 0.392 β

The BMI < normal category (n = 1) was excluded from the statistical analysis due to insufficient sample size and is reported for descriptive purposes only. €: P-value or CI between BMI < normal and BMI = normal; π: P-value or CI between BMI < normal and BMI > normal; β: P-value or CI between BMI = normal and BMI > normal. Bold values indicate statistical significance at P < 0.05. Differences were tested using Mann-Whitney for boys or one-way ANOVA followed by Tukey's post hoc test for pairwise comparisons for girls. CI : Confidence interval. .

Age and sex were simultaneously input as covariates in multiple linear regression models to investigate the independent relationships between motor and executive function outcomes and BMI-for-age Z-score (BAZ), which was considered as a continuous variable (**Table 4**). A modest to good percentage of variance across outcomes was explained by the models (Adj. R2 ranging from 0.155 to 0.626). None of the motor or executive function outcomes were significantly predicted by BAZ (all p > 0.05). On the other hand, age was a reliable and consistent predictor of all seven outcomes (all p < 0.001), which is consistent with preschoolers’ expected developmental trajectory. Inhibition (B = 1.248, 95% CI [1.041, 1.455], p < 0.001), working memory (B = −1.216, 95% CI [−1.406, −1.025], p < 0.001), and upper body strength (B = 0.956, 95% CI [0.067, 1.844], p = 0.035) were significantly associated with sex.

**Table 4:** Multiple linear regression analyzes: associations between BMI-for-age Z-score and motor and executive function outcomes in preschool children, adjusted for age and sex (n = 111).

Outcome	Adj. R²	BAZ		Age (years)		Sex	
		B (95% CI)	p	B (95% CI)	p	B (95% CI)	p
Inhibition	0.574	-0.012 (-0.094, 0.070)	0.778	**0.302 (0.114, 0.491)**	**0.002**	**1.248 (1.041, 1.455)**	**<0.001**
Working memory	0.626	0.004 (-0.072, 0.079)	0.919	**0.359 (0.185, 0.533)**	**<0.001**	**-1.216 (-1.406, -1.025)**	**<0.001**
Functional mobility (s)	0.155	-0.116 (-0.301, 0.068)	0.215	**-0.974 (-1.400, -0.548)**	**<0.001**	-0.411 (-0.878, 0.057)	0.084
Postural steadiness (s)	0.177	0.073 (-1.177, 1.323)	0.908	**7.113 (4.233, 9.993)**	**<0.001**	0.642 (-2.517, 3.801)	0.688
Lower body strength (cm)	0.270	-0.292 (-2.986, 2.403)	0.831	**18.727 (12.521, 24.934)**	**<0.001**	6.435 (-0.372, 13.242)	0.064
Upper body strength (kg)	0.276	0.140 (-0.212, 0.492)	0.432	**2.569 (1.758, 3.379)**	**<0.001**	**0.956 (0.067, 1.844)**	**0.035**
Dexterity (s)	0.391	-0.276 (-1.372, 0.821)	0.619	**-10.279 (-12.804, -7.754)**	**<0.001**	1.679 (-1.090, 4.449)	0.232

B = unstandardized regression coefficient; 95% CI = 95% confidence interval; Adj. R² = adjusted coefficient of determination; BAZ = BMI-for-age z-score (treated as a continuous variable); Sex coded as 0 = girl, 1 = boy. Bold values indicate statistical significance at p < 0.05. All models included BAZ, age, and sex entered simultaneously as predictors.

## 4 Discussion

This cross-sectional observational study provides novel evidence from a North African context on the associations between sex-specific BMI categories, 24-hour MB, MS, and EF in Tunisian preschool children. By combining objective accelerometry with standardized cognitive and motor assessments, the study contributes methodologically rigorous data from an underrepresented population.

Statistically significant differences were primarily observed for sex-specific anthropometric indicators (HAZ, BAZ, weight, and in girls, height and age) across BMI categories, confirming the internal consistency of the classification approach. Regarding 24-hour MB, statistically significant differences were observed for moderate-intensity PA (MPA) in girls. Additionally, among boys, a statistically significant difference was observed in adherence to screen-time guidelines (SST ≤ 60 min/day), with a lower proportion of children in the BMI > normal group meeting the guideline compared with BMI = normal (p = 0.049). No statistically significant differences were observed for light-intensity PA (LPA), vigorous-intensity PA (VPA), moderate-to-vigorous PA (MVPA), total PA (TPA), overall SB, or sleep duration across sex-specific BMI categories.

For MS, no statistically significant differences were observed for most components assessed. However, a statistically significant difference was detected in girls for upper-body strength between BMI = normal and BMI > normal groups (p = 0.003), with girls in the higher BMI category being associated lower performance. Regarding EF (inhibition and visuospatial working memory), no statistically significant differences were observed between BMI categories.

Within the detectable effect sizes of this sample, these findings suggest that, at preschool age, weight status appears to be primarily associated with anthropometric variation, with limited and domain-specific functional differences particularly upper-body strength in girls and adherence to screen-time recommendations in boys. Nonetheless, the absence of statistically significant differences in most variables should not be interpreted as evidence of a lack of potentially meaningful associations.

It should also be noted that the interpretation of the findings should not rely solely on statistical significance. Although most associations did not reach statistical significance (p > 0.05), the observed effect sizes (**Tables 1** and **3**) were generally small to moderate, suggesting limited practical relevance in this sample. However, given the relatively small sample size and the imbalance across BMI categories, the statistical power may have been insufficient to detect small effects, particularly given the subgroup stratification by sex and BMI categories. Therefore, non-significant findings should be interpreted with caution and should not be equated with the absence of true associations.

The convenience sampling, combined with a highly imbalanced distribution of BMI categories particularly the < normal category among boys (n = 1) and girls (n = 4) likely limited the statistical power of sex-stratified analyzes and increased the risk of Type II error. Moreover, the cross-sectional design precludes causal inference. Future research using adequately powered longitudinal and intervention-based designs, with strengthened analytical frameworks, is needed to clarify developmental trajectories related to weight status and to identify mediating and moderating mechanisms underlying the relationships between BMI, MB, and early childhood development.

The absence of statistically significant associations for most outcomes should be interpreted with caution. Given the relatively small sample size and the uneven distribution across BMI subgroups, particularly in the BMI < normal category, the study may have been underpowered to detect small-to-moderate effects. The lack of *a priori* power calculation further limits the ability to determine whether non-significant findings reflect true absence of association or insufficient statistical sensitivity.

Therefore, caution is warranted when extrapolating these findings to other contexts, particularly at the North African level, given potential differences in lifestyle, socioeconomic conditions, and early childhood environments.

### 4.1 BMI and 24-hour movement behaviors

Overall, BMI status did not appear to be strongly associated with objectively measured MB in either boys or girls. Light-intensity PA, MPA, VPA, TPA, sedentary time, and sleep duration did not differ significantly across BMI categories, with the exception of a significant increase (p = 0.040) in MPA among girls with a normal BMI versus BMI above the normal range. These findings are consistent with previous studies showing that, in preschool-aged children, daily movement patterns are largely driven by spontaneous play, environmental opportunities, and developmental stage rather than by body mass or adiposity (Taylor et al., 2018; Bergqvist-Norén et al., 2022). Despite the fact that most children met the MVPA and sleep recommendations, overall compliance with total physical activity (TPA) guidelines remained low. This may be explained by elevated levels of sedentary behaviors, particularly screen time, which likely displace light-intensity physical activity (LPA). In preschool-aged children, LPA represents a substantial component of daily movement; therefore, even when MVPA levels are adequate, reductions in LPA combined with prolonged sedentary time may result in insufficient accumulation of total physical activity over a 24-hour period. Similarly, data from the SUNRISE study in Tunisia have demonstrated relatively homogeneous MB across weight categories in this age group (Ltifi et al., 2025b; Ltifi et al., 2025c).

Evidence from the MENA (Middle East and North Africa) region remains limited (Baddou et al., 2018; Chaabane et al., 2020; Ltifi et al., 2025b), particularly in preschool-aged children, and is characterized by methodological heterogeneity across studies. Available evidence suggests generally low levels of physical activity and high variability in movement behavior patterns across the region, although direct comparisons remain limited due to differences in study designs, measurement methods, and age groups. Systematic evidence further indicates that a relatively small proportion of children in the region meet physical activity recommendations, highlighting a consistent pattern of insufficient activity despite contextual differences. In early childhood populations, available studies also suggest low adherence to 24-hour movement behavior guidelines, reinforcing concerns regarding insufficient total physical activity accumulation. Overall, these limitations highlight the need for more standardized and context-specific research in preschool populations across the MENA context.

Despite the weak association between BMI and MB, overall adherence to WHO 24-hour movement guidelines was low, particularly for TPA and for meeting all recommendations simultaneously. This suggests that insufficient PA and excessive SB affect most children regardless of weight status. An exception was observed for screen-based sedentary time among boys, where those with a BMI above the normal range were less likely to meet the ≤60 min/day recommendation, in line with evidence linking screen exposure to early adiposity through reduced spontaneous activity and increased energy intake (Duch et al., 2013). Such SB may create a feedback loop, where higher BMI reinforces greater screen time, further limiting opportunities for active play and spontaneous movement. Previous studies have also reported that boys often exhibit higher sedentary profiles than girls at preschool age, likely due to preferences for video games and digital content, whereas girls tend to engage more in moderate PA and interactive play (Cheng et al., 2016; Jiang, 2019). These sex-specific differences highlight the importance of early, targeted interventions to limit screen time and promote PA, aiming to prevent excess weight and its potential impact on motor and cognitive development.

### 4.2 BMI and executive functions

No significant differences were observed in inhibition or working memory across BMI categories in either boys or girls. These findings indicate that, at preschool age, weight status is not yet associated with measurable differences in executive functioning. This contrasts with studies in older children and adolescents, in whom overweight and obesity are often linked to poorer executive performance (Mamrot and Hanć, 2019; Martí-Nicolovius, 2022). However, early childhood is characterized by rapid brain development and high neural plasticity, particularly within frontoparietal networks supporting executive control (National Research C and Institute of Medicine Committee on Integrating the Science of Early Childhood D, 2000; Best and Miller, 2010; Diamond, 2013). The biological mechanisms that may link excess adiposity to cognitive dysfunction such as chronic low-grade inflammation, insulin resistance, and altered neurotrophic signaling likely require longer exposure to excess body fat before producing detectable effects (Mamrot and Hanć, 2019; Ltifi et al., 2025a). Moreover, the relatively adequate sleep duration and moderate-to-high activity levels observed in most children may have exerted protective effects on executive development, as both PA and sleep support prefrontal cortex maturation and memory consolidation (Jiang, 2019; Barros et al., 2021; Spencer, 2021). Future longitudinal studies should consider employing additional and more sensitive tools to assess executive function quality, including ecologically valid, task based, or real-world measures, to capture subtle differences that may not be detected by standard inhibition and working memory tasks at this early developmental stage.

### 4.3 BMI and motor skills

Motor performance was largely similar across BMI categories in both boys and girls. Functional mobility, balance, lower-limb strength, and manual dexterity did not differ significantly according to weight status, suggesting that excess body mass does not yet translate into substantial motor limitations at 4–5 years of age. However, an important exception was observed for upper-body muscular strength in girls. Girls with a BMI with-in the normal range showed higher upper-body strength compared with those classified as having a BMI above the normal range. This difference may reflect reduced relative strength due to greater body mass as well as a less favorable ratio of muscle mass to fat mass in girls with higher BMI (Shah et al., 2020; Zorrilla-Revilla et al., 2022). Reduced relative upper-limb strength may limit participation in weight-bearing and climbing-type play activities (e.g., hanging, climbing, object manipulation), which could, over time, contribute to more SB. These findings are consistent with longitudinal studies indicating that the negative impact of excess weight on MS becomes more pronounced with age, as increasing biomechanical load and declining relative strength progressively constrain movement efficiency (Cheng et al., 2016; Lima et al., 2021). Future studies should adopt longitudinal designs and include larger, more diverse populations, while considering broader contextual factors such as environmental influences, lifestyle behaviors, and socioeconomic status. These approaches would enable a more comprehensive understanding of how early life weight status interacts with external determinants to shape motor development trajectories, PA patterns, and long-term health outcomes.

### 4.4 Sex-specific differences

Early childhood development may differ between boys and girls due to biological, behavioral, and environmental factors. Rather than reflecting consistent differences across all domains, the present findings suggest that sex-specific patterns are selective and context-dependent. The observed differences likely reflect early divergences in body composition, neuromuscular maturation, and behavioral preferences, which may influence engagement in specific types of physical activity and motor tasks. For instance, variations in play behavior and interaction with the environment may contribute to distinct developmental pathways already emerging at preschool age. Importantly, these sex-related patterns should be interpreted cautiously, as they may also be influenced by sample characteristics and limited statistical power. Overall, these findings highlight the importance of adopting sex-sensitive approaches in early childhood research. Future longitudinal studies with larger and more diverse samples are needed to better understand how sex interacts with BMI, lifestyle behaviors, and environmental factors to shape motor and cognitive development trajectories (Shah et al., 2020; Bergqvist-Norén et al., 2022; Zorrilla-Revilla et al., 2022).

After adjusting for age and sex, the multiple linear regression analyzes showed that BAZ, when considered as a continuous variable, was not a significant independent predictor of any motor or executive function outcome in preschoolers. These results are in line with an increasing amount of research indicating that the association between early childhood neurodevelopmental outcomes and adiposity may be more complicated than previously thought and may not be fully reflected by anthropometric indices alone (Likhitweerawong et al., 2022). In fact, a number of studies have found similar null associations between BMI-related measures and motor or cognitive performance in preschool-aged children, indicating that factors like sedentary behavior, physical activity levels, and socioeconomic status may be more important mediators than obesity itself (Fernandes et al., 2022).

All seven outcomes showed that age was the most reliable and consistent predictor (all p < 0.001), which is consistent with recognized early childhood developmental trajectories. Due to continued brain growth, myelination, and synaptic pruning, both motor and executive functions mature quickly and significantly during the preschool years (Gilmore et al., 2018; Chichinina et al., 2025). These results highlight how crucial age is as a covariate in developmental studies including preschoolers because neglecting to take into consideration even minor age variations during this time can significantly skew association estimates.

Three results showed variations between the sexes. While girls fared better in working memory, boys did better in inhibition and upper body strength. These results are somewhat in line with the research that has previously documented sex-related variations in the development of executive function during the preschool years, with girls often exhibiting earlier working memory and inhibitory control maturation (Korucu et al., 2022). Further research in bigger and more representative samples is warranted since the direction of the sex impact reported for inhibition in the current study may reflect task-related aspects or sample-specific traits.

### 4.5 Implications for child health

Although BMI was not strongly associated with MB, MS, or EF in preschool children, early childhood remains a critical window for universal preventive interventions. In line with WHO recommendations, it is essential to promote PA, limit SB, and ensure adequate sleep for all children, regardless of weight status (WHO Guidelines Approved by the Guidelines Review Committee, 2019). Integrating structured active play, motor skill development, and screen-time reduction within early childhood settings can support both cognitive and motor development, as well as overall health outcomes (Carter et al., 2011; Tremblay et al., 2017; Okely et al., 2021; Okely et al., 2021). These strategies are particularly important in contexts such as Tunisia (Ltifi et al., 2026), but also in other countries (Sunda et al., 2022; Başarır et al., 2025), where rapid lifestyle transitions may increase early childhood overweight and obesity risk (Cheng et al., 2016; Barros et al., 2021). Therefore, preschool years represent a key period for preventive action, and well-designed educational and environmental interventions can have lasting benefits for children’s physical and cognitive development.

In addition, although not assessed in the present study, dietary, socio-economic, and environmental factors may also play a key role in shaping motor behaviors, physical activity levels, and cognitive and motor development in preschool children within the Tunisian context. Importantly, future studies should incorporate these factors as covariates in multivariate models in order to better isolate the independent associations between BMI, movement behaviors, and developmental outcomes. Furthermore, future research would benefit from multi-country comparative studies across the MENA region to enhance the generalizability and cultural relevance of findings. In addition, the implementation of controlled intervention studies targeting reductions in screen time and the promotion of structured physical activity within preschool settings is warranted to better understand causal mechanisms and inform public health strategies.

### 4.6 Strengths and limitations

This study has several notable strengths. First, it employed objective assessment of 24-hour PA and sleep using accelerometry, which is considered a gold standard method. Second, it utilized sex-specific BMI classifications and standardized assessments of MS and EF, ensuring methodological rigor. Third, the inclusion of children from both urban and rural settings in Tunisia enhances the ecological validity of the findings and better reflects the diversity of living contexts in the region, which remains underrepresented in pediatric movement-behavior research.

However, several limitations should be acknowledged, although children were recruited from both urban and rural kindergartens, the sample was confined to a single geographic region (Greater Tunis) and was based on convenience sampling. As such, the findings may not be fully representative of the national population or generalizable to other North African contexts. Regional disparities in socioeconomic status, cultural practices, and access to physical activity opportunities may influence movement behaviors, motor development, and cognitive outcomes. Therefore, the external validity of the present findings remains limited.

In addition, although multiple regression analyzes were performed with adjustment for age and sex, urban/rural residence was not included as a covariate in the present models, which may represent an additional limitation regarding the control of contextual environmental influences.

The study’s cross-sectional design prevents causal inference, and the absence of an *a priori* power calculation limits the ability to determine whether the sample size was sufficient to detect meaningful effects. In addition, the relatively small and uneven subgroup sizes increase the risk of Type II errors. Some BMI subgroups had small sample sizes, notably the < normal BMI category (n = 1), which was excluded from statistical analyzes and is reported only for descriptive purposes. The small number of children in this BMI subgroup limited statistical power and may have masked subtle associations.

Furthermore, although accelerometry is robust, it may underestimate certain activities, such as cycling or water-based play. The assessment of EF in preschool children also presents developmental constraints, including potential ceiling or floor effects, and the fact that only two EF domains were evaluated may have limited the detection of differences between BMI groups. Additionally, the psychometric properties of the assessment tools, particularly in their Arabic versions, should be considered when interpreting the results.

Finally, dietary intake and other unmeasured factors including genetics, family habits, and socioeconomic environment were not assessed and may partly explain variability in BMI independent of MB. The absence of these dietary, socio-economic, and environmental variables in the present analysis represents an additional limitation and may contribute in part to the variability observed in the results. Future studies should therefore incorporate these factors in order to better understand the complex interactions between BMI, lifestyle behaviors, and child development in similar contexts. Future studies should also extend investigations to multi-country MENA contexts and incorporate family and socioeconomic characteristics to improve external validity and better capture regional diversity.

Therefore, the absence of statistically significant differences between BMI categories should be interpreted cautiously and does not necessarily indicate the absence of meaningful associations.

## 5 Conclusions

This cross-sectional observational study provides novel evidence from a North African context on the associations between sex-specific BMI categories, 24-hour MB, MS, and EF in Tunisian preschool children. By combining objective accelerometry with standardized cognitive and motor assessments, this study offers robust and methodologically rigorous data in a population that remains underrepresented in pediatric research. Statistically significant differences were observed for anthropometric indicators (HAZ, BAZ, weight, and in girls, height and age). Functionally, BMI above normal was observed in association with higher moderate-intensity physical activity (MPA) and lower upper-body strength in girls, while it was observed in association with lower compliance with screen-time guidelines (SST ≤ 60 min/day) in boys. No statistically significant differences were observed for other PA measures, SB, sleep, or EF. It is important to note that the absence of statistical significance should not be interpreted as evidence of absence of meaningful associations, particularly given the limited statistical power and the small and uneven distribution of BMI categories. These results indicate that, in preschool children, BMI is primarily associated with anthropometric variation, with limited but domain-specific functional implications. The small and uneven distribution of BMI categories particularly < normal in boys (n = 1) and girls (n = 4) and the cross-sectional design limit statistical power and preclude causal inference. Given the cross-sectional design of the study, causal relationships cannot be inferred, and the observed associations should be interpreted as correlational. Future longitudinal and intervention-based studies with larger, sex-stratified samples are required to clarify developmental trajectories, and to explore mediating and moderating mechanisms linking BMI, MB, and early childhood functional development.

## Data Availability

The original contributions presented in the study are included in the article/supplementary material. Further inquiries can be directed to the corresponding author.
